# Multi-objective genetic algorithm calibration of colored self-compacting concrete using DEM: an integrated parallel approach

**DOI:** 10.1038/s41598-024-54715-4

**Published:** 2024-02-19

**Authors:** Vahid Shafaie, Majid Movahedi Rad

**Affiliations:** Department of Structural and Geotechnical Engineering, Széchenyi István University, 9026 Győr, Hungary

**Keywords:** Colored self-compacting concrete (CSCC), Genetic algorithm (GA), Automated calibration, UCS test, PFC3D, Multi-objective optimization, Engineering, Civil engineering

## Abstract

A detailed numerical simulation of Colored Self-Compacting Concrete (CSCC) was conducted in this research. Emphasis was placed on an innovative calibration methodology tailored for ten unique CSCC mix designs. Through the incorporation of multi-objective optimization, MATLAB's Genetic Algorithm (GA) was seamlessly integrated with PFC3D, a prominent Discrete Element Modeling (DEM) software package. This integration facilitates the exchange of micro-parameter values, where MATLAB’s GA optimizes these parameters, which are then input into PFC3D to simulate the behavior of CSCC mix designs. The calibration process is fully automated through a MATLAB script, complemented by a fish script in PFC, allowing for an efficient and precise calibration mechanism that automatically terminates based on predefined criteria. Central to this approach is the Uniaxial Compressive Strength (UCS) test, which forms the foundation of the calibration process. A distinguishing aspect of this study was the incorporation of pigment effects, reflecting the cohesive behavior of cementitious components, into the micro-parameters influencing the cohesion coefficient within DEM. This innovative approach ensured significant alignment between simulations and observed macro properties, as evidenced by fitness values consistently exceeding 0.94. This investigation not only expanded the understanding of CSCC dynamics but also contributed significantly to the discourse on advanced concrete simulation methodologies, underscoring the importance of multi-objective optimization in such studies.

## Introduction

Self-compacting concrete (SCC) and colored self-compacting concrete (CSCC) have gained significant attention in the construction industry due to their unique properties and wide range of applications. The design of SCC requires a careful balance of material components to achieve workability, flow without segregation, and stability, considering factors like yield stress and plastic viscosity^1^. SCC's development has significantly improved construction durability while self-compacting mortars (SCMs) offer enhanced filling capability in congested structures, resulting in cost and labor reduction for concrete repair and rehabilitation^1–6^. Incorporating pigments into SCC transforms the appearance of concrete structures, providing a range of colors and textures and opening up new architectural possibilities^7–9^. CSCC combines the fluidity and filling capacity of SCC with the aesthetic appeal of traditional colored concrete, providing versatile options for intricate architectural designs^1,10^. The examination of the performance attributes of CSCC and its potential applications in the field of civil engineering offers a promising pathway for continued investigation and exploration.

Following the advancements in the realm of SCC and CSCC, the intricate balance between their components and their broad applications has indeed streamlined construction procedures and broadened architectural horizons^1,7,9^. However, the key to understanding these materials on a granular level and predicting their behaviors under varying conditions lies in the realm of numerical simulation. The Discrete Element Method (DEM) has rapidly become an indispensable tool for analyzing the intricate mechanical behaviors of a range of materials, encompassing rocks, concretes, and other composite substances. Within the vast ambit of rock mechanics, PFC, as a DEM-oriented software, demands precise calibration to ensure its simulations align with real-world conditions. The significance of such calibration is highlighted, noting that even minute deviations in micro-parameters can have profound implications on the outcomes of numerical simulations, especially in physical experiments like unconfined compression tests^11^. The continuous evolution of DEM methodologies has led to the introduction of innovative models. A prime example is the Weibull linear parallel bond model^12^. This model presents a paradigm shift in how heterogeneous rocks are simulated, offering an efficient means to study their nonlinear mechanical behaviors. The continued development in this domain accentuates the critical importance of micro-parameter calibration. It became imperative for simulations to mirror the intricacies of real-world scenarios, ensuring accuracy and reliability in predictions. The detailed investigations by^13,14^ further emphasize the nuances and complexities inherent to material interfaces and the pivotal role of boundary conditions in influencing outcomes. Such research endeavors, combined with the ongoing evolution of DEM methodologies, highlight the field's commitment to achieving unparalleled precision and robustness in simulations.

In the foundational stages of DEM calibration, the primary method was rooted in trial-and-error techniques^15^. This hands-on, heuristic approach necessitated an iterative cycle of adjusting unknown input parameters to align DEM simulated outcomes with observed bulk behaviors. While seemingly straightforward, this method encountered challenges. The innate simplicity of this approach was constrained by the multi-dimensional nature of parameters and the computational rigor required for comprehensive DEM simulations^16^. In the evolving realm of DEM calibration, a multitude of optimization techniques have been deployed to ensure accuracy. The Levenberg–Marquardt method, known for its aptitude in residual minimization^17^, is complemented by techniques such as the Nelder-Mead simplex^18^ and the weighted least squares approach^19^. Other prominent strategies include the Gauss–Newton algorithm^20^, enhanced simulated annealing algorithm^21^, Differential Evolution (DE) algorithm^22^, and Particle Swarm Optimization (PSO)^23^. Notably, genetic algorithms have gained traction, highlighting the wide array of computational approaches leveraged in this field^16,23,24^.

In the realm of engineering optimization, the genetic algorithm (GA), rooted in the principles of natural selection and species evolution, emerges as a robust and adaptable tool^25,26^. As a meta-heuristic technique, the GA is renowned for its efficacy in resolving complex, multi-modal, and sizable problems, often yielding satisfactory rather than precise solutions. Characterized as a probabilistic method, the GA is capable of producing diverse outcomes from identical initial conditions, thereby generating high-quality solutions in multi-objective scenarios. The applicability of GAs is influenced by various factors including problem complexity, constraint types, variable nature, and objective functions. While advantageous in exploring extensive and intricate search spaces, GAs are recognized for their potential computational intensity and variable performance contingent upon specific problem parameters^27–29^. Despite these limitations, the utility of GAs in engineering, where a balance between solution accuracy and computational efficiency is paramount, remains undisputed.

When confronting scenarios that require consideration of multiple bulk properties, the analytical task often transitions into a multi-objective optimization problem (MOOP)^16^. Delving into the history of computational strategies, multi-objective evolutionary genetic algorithms (MOEAs)^16,24,30–33^ have consistently showcased their effectiveness in navigating these intricate challenges. Building on this foundation, the present study introduces an advanced DEM calibration approach. By integrating MATLAB's Genetic Algorithm (GA) with PFC3D software, we have formulated an automated, concurrent, and iterative procedure adeptly tailored for calibrating the micro-parameters of ten unique CSCC mix designs. This systematic approach ensures congruence between PFC3D simulations and observed macro properties, offering insights into the intricate behaviors of CSCC and setting a precedent for subsequent PFC3D simulations. The experimental basis of our research is fortified by the insights from^34^, providing a deep dive into the interfacial bond strength of colored SCC repair layers. By integrating such foundational data with cutting-edge numerical methodologies, as exemplified by the differential evolution calibration method by^22^, our research stands at the nexus of traditional experimentation and contemporary computational techniques.

What sets this research apart is its novel approach to calibration. Our work introduces a fresh perspective to the field, particularly by embracing multi-objective optimization and deploying an automated parallel calibration process. Unlike typical practices in rock mechanics or geomechanics, where both triaxial and uniaxial tests are conducted together for calibration, the focus in this study is specifically placed on the uniaxial compressive strength (UCS) test for calibrating CSCC models. This approach, tailored to the context of concrete, emphasizes the UCS as a standard test, diverging from the broader range of tests often utilized in rock or geomechanical studies. The calibration of the Poisson's ratio, based on UCS test results and considering both axial and lateral strain measurements, is addressed. This aspect of the calibration process highlights the adaptation and refinement of traditional methods to suit the specific properties of CSCC. This research is also pioneering in its focus on CSCC, especially by factoring in the pigment's influence on cohesive micro-parameters, a feat seldom explored in previous studies. Furthermore, the concurrent calibration across ten distinct mix designs accentuates the depth and complexity of our approach. In culmination, the primary aim of this paper is to present a refined DEM calibration framework, emphasizing the novelty of our approach in multiple dimensions, from the integration of advanced optimization techniques to the detailed exploration of CSCC behaviors. As the research progresses, it becomes imperative to acknowledge the symbiotic relationship between computational methods and hands-on experimentation, emphasizing that one complements the other, enriching our understanding of materials like CSCC.

The structure of this paper is delineated as follows: Section "Materials and methods" elucidates the 'Materials and Methods' employed; Section "Results and discussions" presents 'Results and Discussions', drawing upon the findings and their implications; and Section "Conclusion" concludes the study, summarizing key outcomes.

## Materials and methods

The baseline experimental data and methodologies underpinning the present research are detailed herein. Initially, a revisit to a prior experimental investigation on CSCC repair layers is made, detailing the specific mix designs and their associated uniaxial compressive strength (UCS) test results. These experimental findings serve as the cornerstone for the subsequent numerical study. Following this, the numerical modeling approach, employing the PFC3D software, is expounded upon, detailing the calibration and validation processes set against the experimental results. This methodological framework ensures a thorough exploration of the interfacial bond strength of CSCC repair layers, bridging both experimental and numerical perspectives.

### Experimental description of interfacial bond strength of coloured SCC repair layers

The foundation for the current numerical study is based on the findings from a preceding experimental investigation into the interfacial bond strength of CSCC repair layers^34^.

#### Mix designs from the experimental study

In the referenced investigation, ten unique SCC mixes were meticulously formulated and evaluated. Mix 1 (Fa10) served as a control mix devoid of any pigments. Mixes 2 through 10 incorporated pigments—namely green, red, or blue—at levels of 5%, 10%, or 15% by weight, replacing the cumulative weight of cement and fly ash^34,35^. The entirety of these mix designs form the basis for the present numerical analysis.

#### Specimen details and UCS results

**Figure 1:**
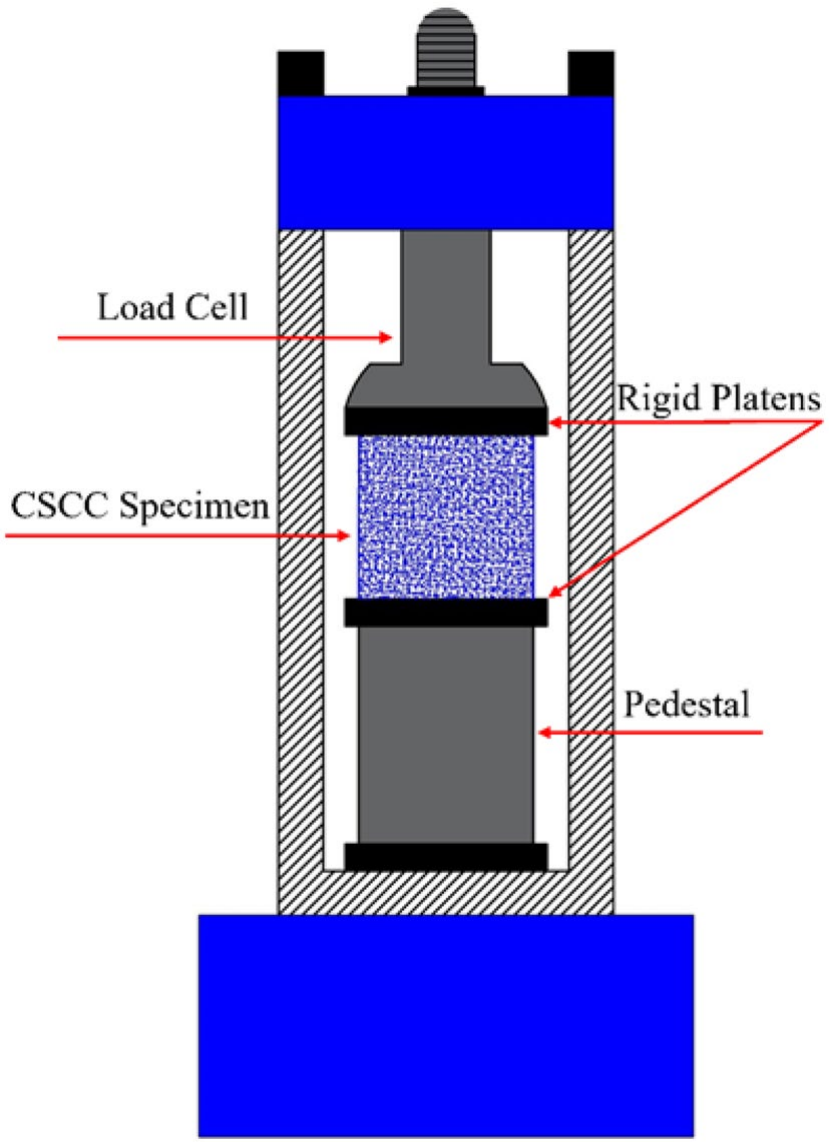
Schematic UCS test setup for CSCC.

For the experimental investigation, cubic specimens of dimensions 15 cm × 15 cm × 15 cm were prepared. These samples were subjected to UCS tests across all ten mix designs (Fig. 1), providing essential data on the stress–strain relationships and the characteristic compressive strengths of each mix. These details are critical for the subsequent numerical modeling. The experimental data revealed a decrease in compressive strength for all SCCs with pigment addition. This reduction in strength correlated with the percentage of pigment replacement. Specifically, with a 10% replacement of cement and fly ash by weight, the compressive strengths of blue, green, and red SCCs decreased by 14%, 18%, and 42%, respectively^34^.

### Fundamentals of DEM modelling with PFC3D

The discrete element method (DEM) is a numerical method used to simulate the behavior of granular materials, such as sand, gravel, and concrete^36^. DEM modeling is based on the concept of treating each particle as an individual entity that interacts with other particles and the surrounding environment. Numerical simulation is a more advanced analysis method based on computer resources, and it is different from the traditional laboratory test method. The DEM is used to solve the problems of discontinuous media by analyzing the interlocking contacts of discrete elements^37^. PFC3D is a software package developed by Itasca that uses the DEM method to simulate the behavior of granular materials^38,39^.

To replicate the behavior of bonded or cohesive materials like concrete, PFC3D employs the Parallel Bonded Particle Model (PBPM). This model incorporates various contact models and has been endorsed for its capability to emulate the macroscopic characteristics of such materials, as well as shed light on the micromechanical phenomena underpinning these behaviors^38,40^. Considering the cemented nature of the experimental specimens used in this study, the Parallel Bonded Contact Model (PBCM) is the chosen approach.

#### Parallel bonded particle model (PBPM)

PFC3D uses a bonded particle model to simulate the behavior of granular materials. The bonded particle model is a type of DEM model that represents the particles as a collection of bonded particles. The bonds between the particles are modeled as springs, which can deform and break under different loading conditions^39^. The parallel bond model is a type of bonded particle model that can be used to simulate the behavior of materials that undergo significant deformation, such as concrete^38,40^. A parallel bond can be envisioned as a set of elastic springs with constant normal and shear stiffnesses uniformly distributed over a disk in 3D. The parallel bond model can transmit both a force and a moment, while the contact bond model can transmit only a force^39,40^.

**Figure 2:**
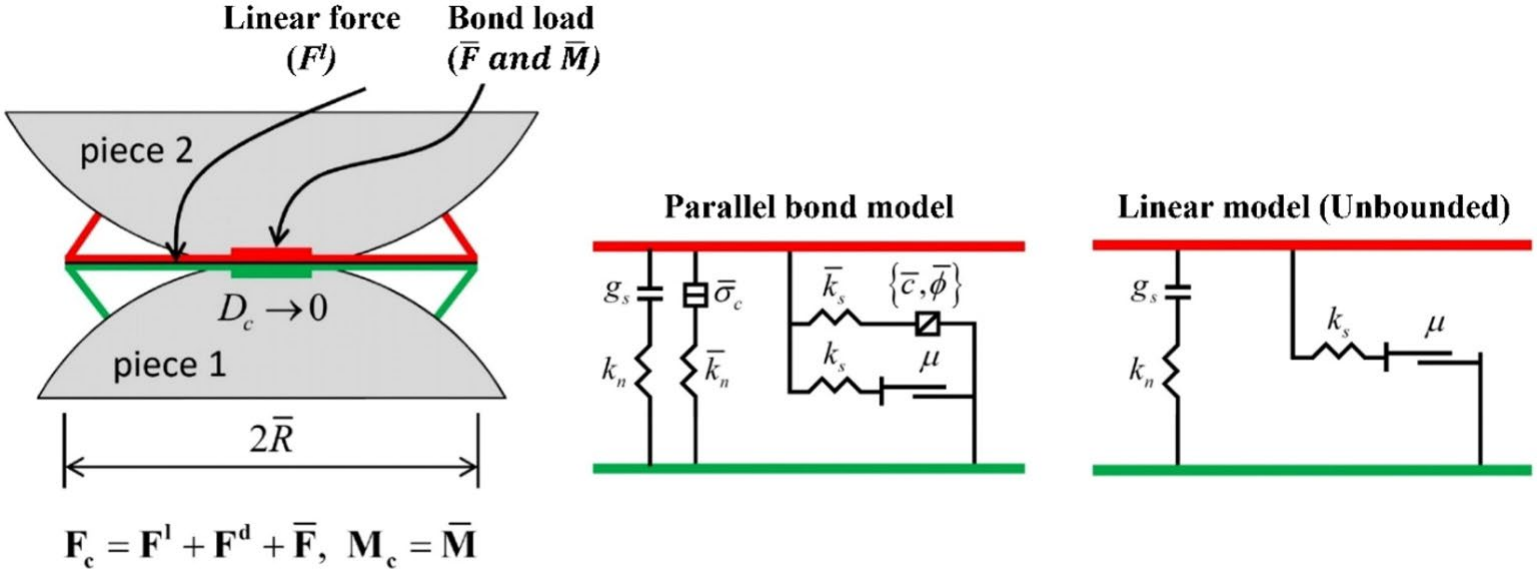
Schematic diagram of LPBM's structure (modified from^39,41^).

The linear parallel bond model (LPBM) delineates two interaction interfaces: a minuscule, tension-free channel and a broader, bonded channel, both bearing distinct forces (Fig. 2). The former mirrors a standard linear model, showing no resistance to relative rotation shifts and adhering to a Coulomb shear force limit. The latter, termed the "parallel bond", aligns with the initial interface when bonded, resists relative rotation, and maintains linearity until a strength threshold breaks the bond. As the material reaches its conclusive phase, bonds are established at grain-grain contacts where the gap is equal to or narrower than the installation gap (${g}_{i}$*)*. The normal and shear stiffnesses are configured based on designated deformability parameters, specifically ${\overline{E} }^{*}$ and ${\overline{k} }^{*}$, as described in the parallel bond deformability method. Concurrently, the remaining characteristics of the second interface are congruent with those outlined in the parallel-bond subset of the linear parallel bond model^39,41^.In its unbonded state, it bears no load. This configuration, when paired with inactive dashpots and a null reference, aligns with Potyondy and Cundall's conceptualization^38^.

In Fig. 2, part (a) presents a detailed depiction of the parallel bonded interface. Here, ${F}_{c}$​ signifies the aggregate force exerted at the contact interface. Other forces including ${F}^{l}$, ${F}^{d}$ and $\overline{F }$ correspond to the linear, damping, and parallel bonded forces, respectively. Meanwhile, part (b) showcases the operational dynamics and associated rheological constituents of the linear parallel bond model.

Given the intricate nature of CSCC, with its unique blend of materials and properties, leveraging the Parallel Bonded Particle Model in PFC3D becomes indispensable. The detailed granular representation and bond mechanics provided by this model ensure a more realistic simulation of the concrete's behavior under varied conditions. As deeper insights into the composition of CSCC are gained, special consideration must be accorded to the incorporated pigments. These not only enhance the aesthetic appeal of concrete but also significantly contribute to its mechanical properties due to their cementitious nature^34^. Within the framework of the Parallel Bonded Particle Model, these pigments are envisioned as integral components of the cemented matrix, interacting both at inter-particle levels and with other concrete constituents. Such interactions can be effectively captured by the bond mechanics of the model, underscoring the synergy between aesthetics and structural integrity, thereby paving the way for an enhanced understanding and potential optimizations of CSCC.

### Numerical model setup

**Figure 3:**
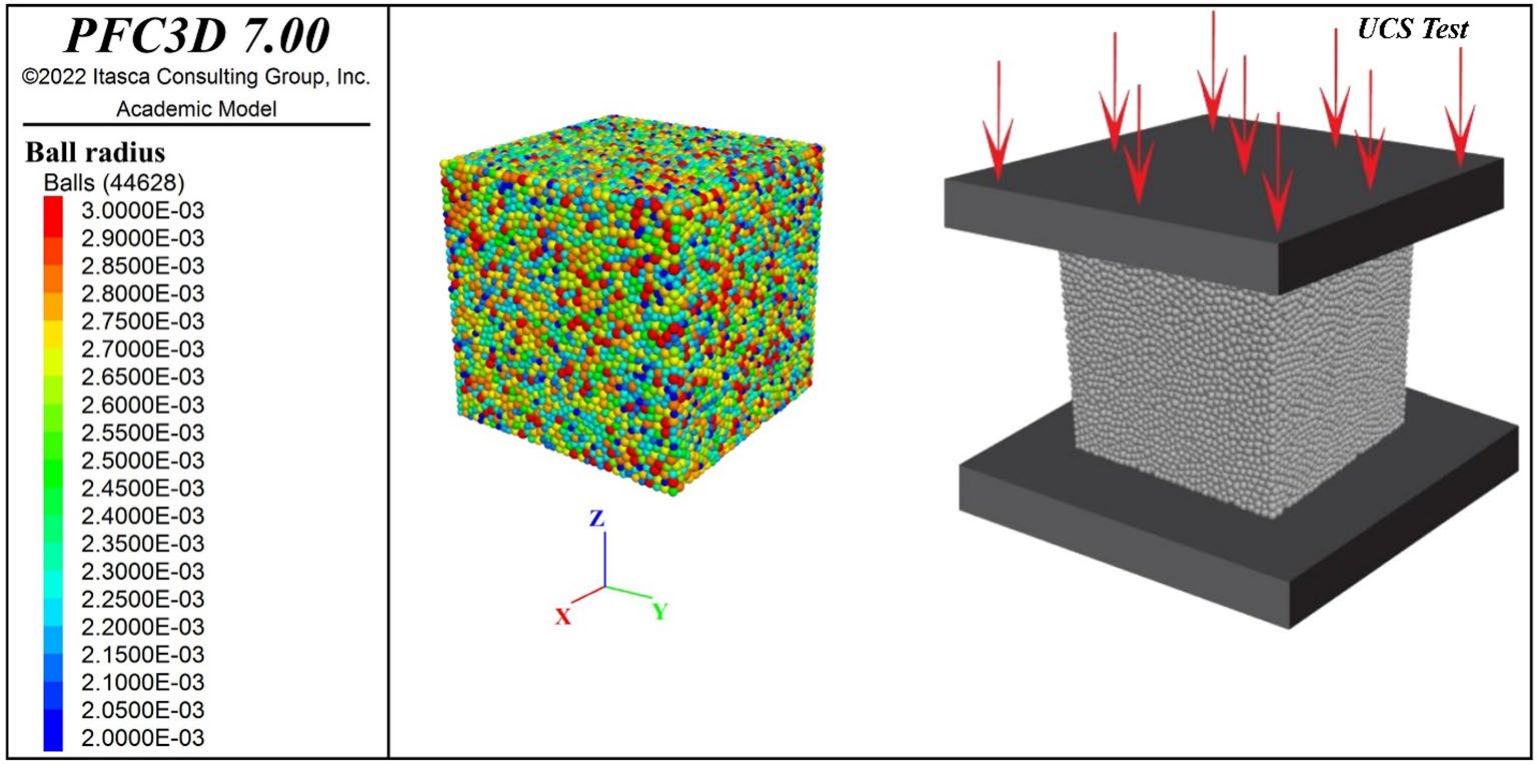
PFC3D model setup; UCS test.

The present study employed PFC3D version 7.0 to devise a comprehensive numerical model, drawing upon experimental findings from ten distinct mix designs detailed earlier. Central to this approach, within the context of a homogeneous model, was the meticulous calibration of micro-parameters, strategically aligned with their corresponding macro-parameters of CSCC. The calibration process heavily relies on the UCS test, a cornerstone in PFC3D concrete modeling, specifically tailored to address the unique properties of CSCC and diverging from the broader range of tests (triaxial, biaxial,..) typically used in rock or geomechanical studies. MATLAB's Genetic Algorithm (GA) was integrated with PFC3D to streamline and optimize the calibration, enabling an automated, iterative optimization of the micro-parameters across the ten unique CSCC mix designs. During simulations, the loading platens, which mimic diametral compression, are represented using rigid plate walls. The top wall is set to a constant velocity of V = 0.01 m/s, while the bottom plate remains static (Fig. 3).

#### Model geometry and boundary conditions

The fundamental properties of CSCC cubic specimens based on comprehensive literature reviews and sensitivity analyses are initialized in this phase. A model size of a 0.15 m cube is established, with ball properties accordingly applied. Particle size distribution is managed by setting both upper and lower particle radius limits, with the sizes then uniformly distributed across this range. Therefore, the ratio of the maximum to minimum particle radius ($\frac{{R}_{max}}{{R}_{min}}$) serves as a more relevant parameter to effectively capture the influence of particle size distribution^42^. Following the literature review, the preliminary sensitivity analyses were conducted to refine our choice of particle sizes^11–14,43–46^. These analyses were crucial in determining how different particle size distributions impact the macro-level properties of CSCC in our simulations. Based on this, a minimum radius (${R}_{min}$= 0.002 m) and a ratio of maximum to minimum radius ($\frac{{R}_{max}}{{R}_{min}}$ = 1.5) were selected, ensuring that these values optimally represent the aggregate distribution within CSCC while maintaining computational efficiency. The variation coefficients for the three primary macro-properties were found to escalate as the specimen heterogeneity increases with the radius ratio. It is advisable to maintain the radius ratio within 1 to 2 to attain a satisfactory coefficient of variation, ideally around 5%^21,46,47^. Ball density is set at 2600 kg/m^3^, aligned with the aggregate properties from experimental tests^34^. Table 1 presents the model geometry micro-parameters, which remain constant for all ten mix designs. This foundation ensures that the subsequent simulations are grounded on realistic configurations.

**Table 1:** Model geometry’ micro-parameters; constant for all ten mix designs.

Model geometry and boundary conditions	
$\mathrm{Model height}, H(m)$	0.15
$\mathrm{Model width}, W (m)$	0.15
${\mathrm{Minimum radius}, R}_{min} (m)$	0.002
$\frac{{R}_{max}}{{R}_{min}}$	1.5
${\text{Porosity}}$	0.1
$\mathrm{Ball density}, \rho$ (kg/m^3^)	2600
$\mathrm{Damping coefficient}$	0.5

#### Static LPBM Micro-parameters

Leveraging prior research and preliminary sensitivity analysis, pivotal micro-parameters, including ${{\varvec{g}}}_{{\varvec{i}}}$, ${\varvec{\phi}}$, ${\varvec{\mu}}$ are pre-defined to their optimal values, offering a consistent baseline across all ten specimens^11–14,21,43,44,46,48^. This strategic fixity, like the adoption of a 35° friction angle (ϕ), was instrumental in minimizing iteration time while maximizing calibration, accuracy, ensuring a more streamlined calibration process for the other six micro-parameters (Table 2).

**Table 2:** Static micro-parameters; adapted for all ten mix designs.

Parallel bond micro-parameters	
$\mathrm{Installation bond gap},{g}_{i} (m)$	5.0e−5
$\mathrm{Friction angel}, \phi (^\circ )$	35
$\mathrm{Friction coefficient}, \mu$	0.7

### Algorithmic optimization of CSCC micro-parameters calibration

Achieving an accurate and robust numerical model depends on the precise calibration of micro-parameters to match the macroscopic properties observed in experimental studies. A classic Multi-Objective Genetic Algorithm (MOGA) approach within MATLAB scripting, and the PFC3D fish scripting facilitated this automated calibration process. In this research, MATLAB's Genetic Algorithm (GA) was integrated with the PFC3D software, creating an automated and iterative optimization process for calibrating the micro-parameters of ten distinct CSCC mix designs. The calibration methodology, grounded on a sequence of systematic steps, was designed to ensure convergence between the simulations in PFC3D and the observed experimental macro properties. Initially, MATLAB launched PFC3D simulations using a set of initial parameters. During these simulations, PFC3D generated output data, like stress–strain profiles, which were stored in designated files and directories. MATLAB then extracted this data, marking the beginning of a crucial multi-objective optimization phase. In this phase, the Genetic Algorithm optimized the numerical macro parameters based on the data from PFC3D. Following the optimization, MATLAB modified the parameter input files for PFC3D with the optimized values, leading PFC3D to perform new simulations with these updated parameters. This iterative calibration process, essential for aligning micro-parameters with experimental benchmarks, was managed within MATLAB. It involved assessing the newly generated numerical macro parameters against experimental data using fitness value calculations. Parameters meeting the optimization targets were considered validated, and their associated micro-parameters were calibrated accordingly.

The calibration continued with MATLAB checking the termination criteria for each of the 10 CSCC mix designs after every iteration. MATLAB initiated another iteration loop otherwise, when not all termination criteria for the 10 CSCC mix designs were met. This loop involved adjusting micro-parameters based on Genetic Algorithm operations including mutation, crossover, and selection, after which the adjusted micro-parameters were used to update PFC3D's input files for the subsequent run. This process repeated iteratively until the termination criteria for all mix designs were satisfied, showcasing a comprehensive and efficient calibration loop that effectively exchanges information between MATLAB and PFC3D.

**Figure 4:**
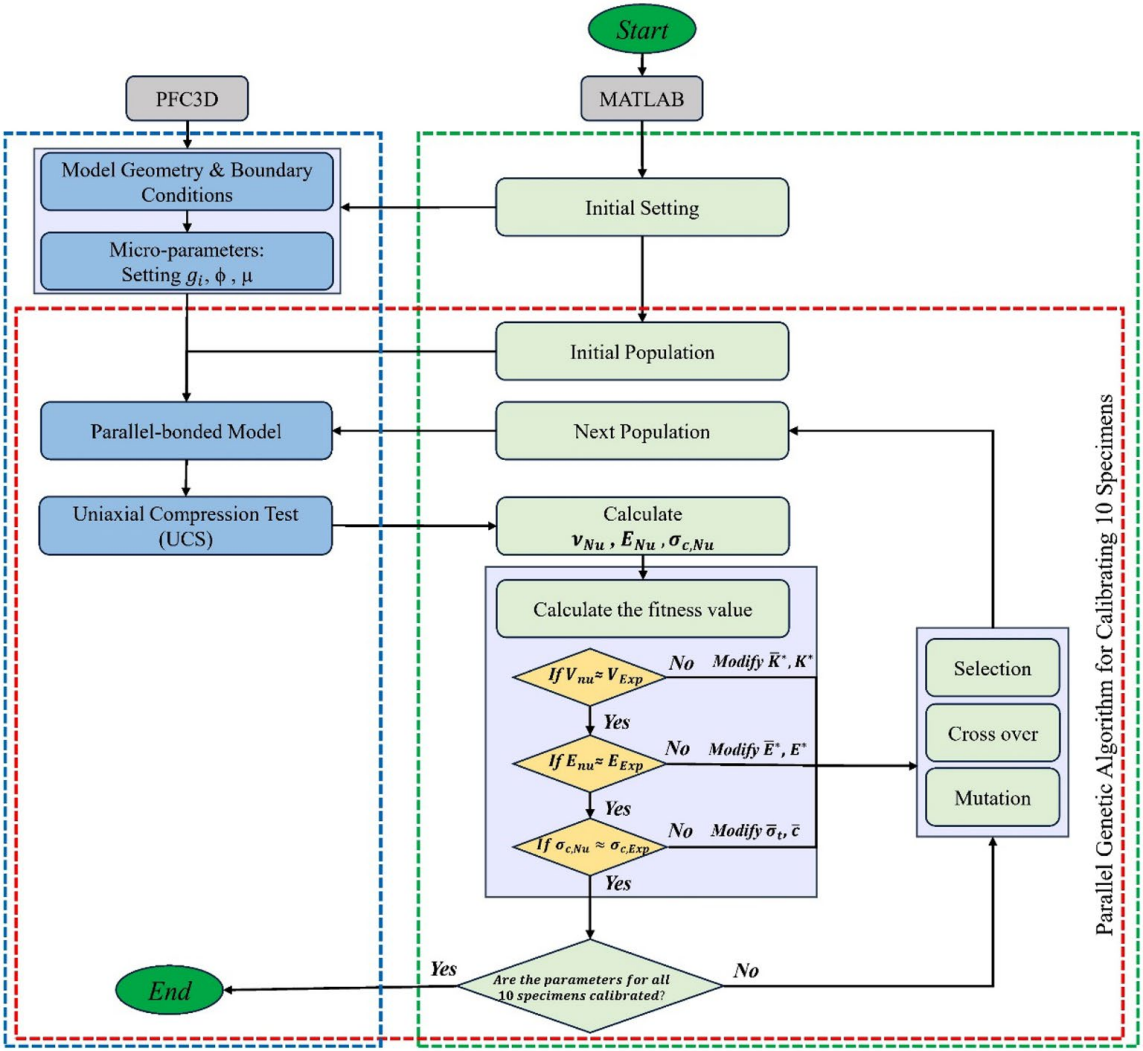
Flowchart of calibrating micro-parameters of CSCC via parallel genetic algorithm.

Building on this structured approach, the numerical model was adeptly fine-tuned for CSCC simulations in PFC3D, capturing the intricate behavior of the unique mix designs. Figure 4 presents the flowchart illustrating the iterative calibration of concrete specimen microparameters, showcasing the integrated optimization dynamics between PFC3D and MATLAB.

#### Optimization framework

In recent decades, the genetic algorithm (GA) has emerged as a noteworthy method for optimizing structural performance. Rooted in the principle of 'Survival of the Fittest', GA operates on a population of chromosomes, applying genetic operators such as mutation and crossover to introduce variations in each computational iteration^49^. It has been highlighted that the inherent nature of certain research problems necessitates the consideration of diverse objective functions. In such contexts, the adoption of multi-objective optimization becomes indispensable, ensuring a more comprehensive calibration approach that addresses the multifaceted objectives intrinsic to the study^16,24^. Precision in defining objectives and understanding design needs are paramount. In this paper, multi-objective optimization has been employed to minimize the mean squared errors (MSE) in the six micro-parameters of the parallel bond (${\overline{{\varvec{K}}} }^{\mathbf{*}}{,{\varvec{K}}}^{\mathbf{*}}$**, **${\overline{{\varvec{E}}} }^{\mathbf{*}}$**, **${{\varvec{E}}}^{\mathbf{*}}$**, **$\overline{{{\sigma }}_{t}}$**,** $\overline{{\varvec{c}} })$ in PFC3D. This objective function is designed to ascertain optimal values for the ten CSCC mix designs, emphasizing the direct or indirect influence of design variables on the function.

#### Calibration process

Building on the GA framework, MATLAB, serving as a control-based system, orchestrates the calibration process in tandem with PFC3D. Upon defining the initial configurations, MATLAB sends commands to PFC3D to initiate the model based on these settings. Once established, the calibration undergoes iterative refinement, with MATLAB overseeing and controlling each iteration. Throughout this process, selections are made based on the fitness values, reflecting the ongoing convergence of simulations toward the experimental macro properties. Calibration terminates when the MSE for the 10 mix designs aligns with the experimental test properties, signaling the genetic algorithm to cease and report the optimal micro-parameters. In this paper, multi-objective optimization has been utilized to minimize the mean squared errors (MSE) or maximize the fitness values of three numerical macro parameters (${{{\varvec{\nu}}}_{{\varvec{N}}{\varvec{u}}} \, \text{,} {\varvec{E}}}_{{\varvec{N}}{\varvec{u}}} , {{\varvec{\sigma}}}_{{\varvec{c}},{\varvec{N}}{\varvec{u}}}$) resulting in the calibration of the six microparameters $({\overline{{\varvec{K}}} }^{\mathbf{*}}{,{\varvec{K}}}^{\mathbf{*}}$**, **${\overline{{\varvec{E}}} }^{\mathbf{*}}$**, **${{\varvec{E}}}^{\mathbf{*}}$**, **$\overline{{{\sigma }}_{t}}$**,** $\overline{{\varvec{c}} })$ of the parallel bond in the PFC3D. The defined objective functions are multi-faceted and parallel to determine the optimal values for the ten CSCC mix designs. Three objective functions have been employed as described below:

##### Objective functions

Upon the determination of the numerical values for ${{{\varvec{\nu}}}_{{\varvec{N}}{\varvec{u}}} \, \text{,}\boldsymbol{ }\boldsymbol{ }\boldsymbol{ }{\varvec{E}}}_{{\varvec{N}}{\varvec{u}}}\boldsymbol{ },\boldsymbol{ }{{\varvec{\sigma}}}_{{\varvec{c}},{\varvec{N}}{\varvec{u}}}$, they are contrasted against the respective experimental benchmarks using objective functions (Eq. 1). Should a high degree of congruence or approximation between these metrics emerge (Fitness value $\ge 0.9$), the corresponding micro-parameters are deemed as calibrated. With this affirmation of calibration, the iterative method unfolds, centering on the calibration of the remaining mix designs. This iterative repetition refines the simulated macro parameters, drawing them closer to the target metrics.

1 $$Objective\, Functions=\left\{\begin{array}{c}Min \left(MSE\right) \equiv Max \,Fitness \,value\left\{If\begin{array}{c}\begin{array}{c}{\nu }_{Nu}\approx {\nu }_{Exp}\\ {E}_{Nu}\approx {E}_{Exp}\end{array}\\ {\sigma }_{c,Nu}\approx {\sigma }_{c,Exp}\end{array}\right.\\ Fitness \,value \ge 0.9 \leftrightarrow Calibration\, boundary \\ 0\le Fitness\, value \le 1.0\end{array} \right.$$

Utilizing these objective functions as a basis for comparison, the structured calibration process enriches the numerical model for CSCC simulations in PFC3D, capturing the intricate behavior of the specific mix designs. Subsequent sections delve deeper into the specific steps of the optimization.

##### Initial settings and value assignment

The foundational step in our simulation workflow involves initializing the model geometry and boundary conditions to ensure consistency throughout the simulations. Simultaneously, initial values for six parallel bond micro-parameters are strategically determined from literature, ensuring an efficient and informed starting point. Alongside, three other static parallel bond micro-parameters (${{\varvec{g}}}_{{\varvec{i}}}$, $\phi$, $\mu$) are designated fixed values within MATLAB, enhancing the calibration process's efficiency. Recognizing the influential role of pigments in the CSCC on the mechanical properties of concrete, their effects are methodically incorporated at this stage, ensuring a holistic representation of the CSCC's behavior.

Furthermore, experimental macro parameters (${{{\varvec{\nu}}}_{{\varvec{N}}{\varvec{u}}} \, \text{,} {\varvec{E}}}_{{\varvec{N}}{\varvec{u}}} , {{\varvec{\sigma}}}_{{\varvec{c}},{\varvec{N}}{\varvec{u}}}$) from laboratory UCS tests across all ten mix designs are cataloged within MATLAB, resulting in a dataset of 30 reference records. After these meticulous adjustments and assignments, the parameters for the genetic algorithm are set as outlined in Table 3. MATLAB, with these parameters in place, crafts the initial population, instigating the first iteration and prompting PFC3D to run a model rooted in these initial values.

**Table 3:** Parameters of the genetic algorithm.

Parameters	
*Population size*	20
*Crossover probability*	0.7
*Mutation probability*	0.3
*Number of crossover offspring*	$2\times (Round\left(Crossover \,probability \times \frac{Population\, size}{2}\right))=14$
*Number of mutation offspring*	$Round \left(Mutation \,probability \times Population \,size\right)=6$
*Maximum of variables (Max_var)*	${\overline{{\varvec{K}}} }^{*}{, {\varvec{K}}}^{*}$= 3.0
	${\overline{{\varvec{E}}} }^{*}$, ${{\varvec{E}}}^{*}$ = 1e11
	${\overline{{\varvec{\sigma}}} }_{{\varvec{t}}}$, $\overline{{\varvec{c}} }$ = 1e8
*Minimum of variables (Min_var)*	${\overline{{\varvec{K}}} }^{*}{, {\varvec{K}}}^{*}$=1.0
	${\overline{{\varvec{E}}} }^{*}$, ${{\varvec{E}}}^{*}$= 1e8
	$\overline{{{\sigma }}_{t}}$, $\overline{{\varvec{c}} }$ = 1e5
*Maximum number of iterations*	50

##### Population size

The size of the population is one of the most crucial parameters for evolutionary algorithms. Proper configuration aids significantly in reducing computational load and decreasing the execution time of an algorithm^50,51^. Considering the high computational overhead for evaluating each population in this study, the population size has been set at 20.

##### Crossover

The crossover operator is used to recombine two chromosomes, aiming to produce superior chromosomes. During the genetic algorithm's crossover operation, the genetic material of two chromosomes from the previous generation's population crossover results in new chromosomes in the current generations^49^. In other words, the recombination process mixes genes present in two chromosomes, thereby producing new chromosomes in the current population. The percentage of the population subjected to crossover is crucial. Usually, a larger percentage of the offspring population is produced through crossover. Furthermore, within DEM material calibration, distinct micro-parameters hold varying degrees of significance. While some parameters operate independently, others are intricately interlinked. As a result, designating a crossover strategy for DEM calibration does not squarely fit into traditional lower or upper crossover frameworks^22^. Therefore, in this study, 70% of the generated population will be created by crossover.

##### Mutation

The mutation operator is considered one of the most essential evolutionary processes to achieve an optimal solution in the genetic algorithm^52^. In the mutation operation, new information is added to the search process in the genetic algorithm randomly. Given that mutation naturally occurs infrequently, the percentage of the mutated population is set at 30% of the initial population.

##### Selection

One of the common and widely used operators for selecting from the generated populations is the Roulette-Wheel Selection method^49^. In this operator, the probability of selecting a chromosome is calculated proportional to its fitness. Therefore, the likelihood of selecting the i-th chromosome will be proportional to its fitness.

##### Parameter range strategy

To optimize computational efficiency and enhance accuracy, a strategic approach was adopted for defining the range of the six micro-parameters. Instead of relying on an inherently broad range from zero to an indefinite value, we established a more focused and intelligent range. This decision was informed by a thorough investigation of prior research related to concrete or synthetic rock, coupled with sensitivity analyses^14,21,22^. Such a strategic range not only facilitates MATLAB's intelligent selection within a contextually relevant span but also ensures convergence of the algorithm and the practical applicability of the solutions. The specific minimum and maximum values defining this range for each micro-parameter can be found in Table 3.

##### Number of iterations and termination condition

The termination criteria of an algorithm play a pivotal role in determining its execution time. It has been observed that initially, the progress of the genetic algorithm in solving a problem is quite commendable, and better solutions are achieved with each iteration. However, in later stages or subsequent iterations, only marginal improvements are observed. In this study, the iteration of the genetic algorithm will continue until the value of the objective function reaches a predefined threshold. By default, a value of 50 iterations has been set for this purpose. If the predefined conditions for achieving the optimal solution are met before 50 iterations, the execution of the algorithm will halt.

##### Calculate numerical macro parameters

**Figure 5:**
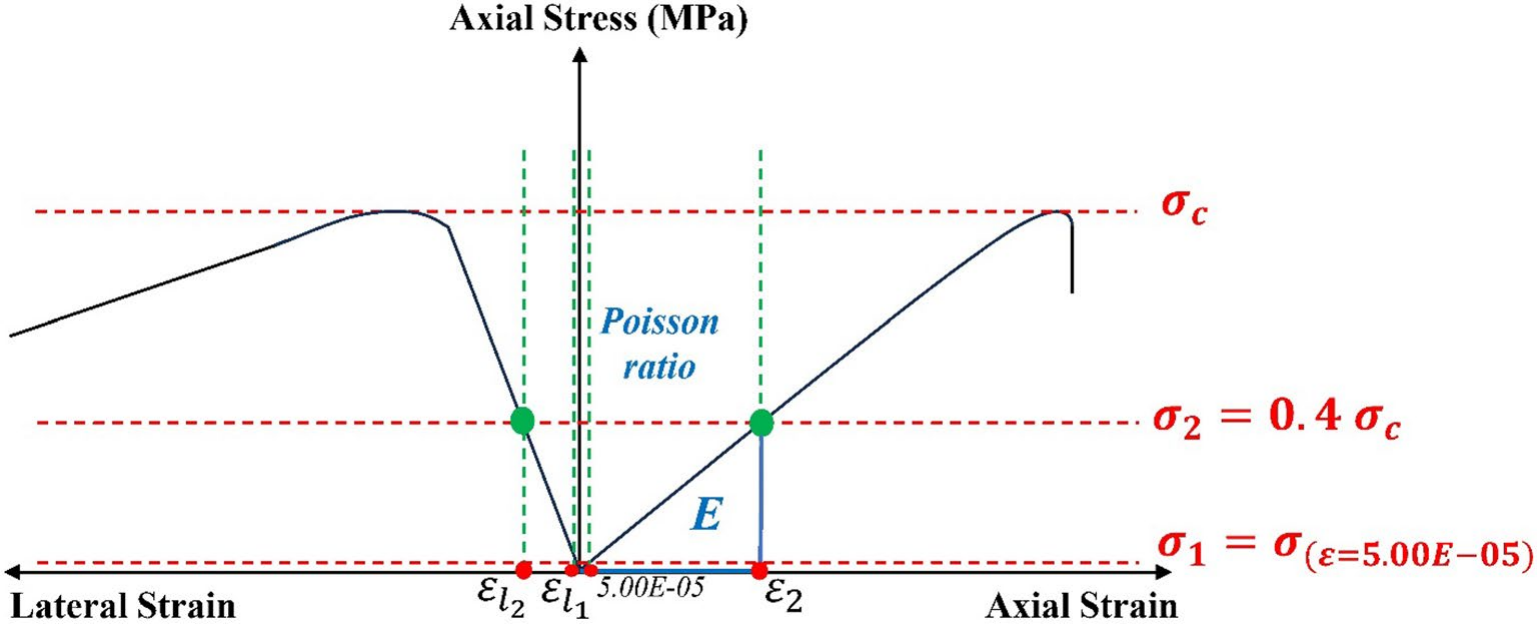
Calculation of ${\upnu }_{{\text{Nu}}}{ ,{\text{E}}}_{{\text{Nu}}}{ ,\upsigma }_{{\text{c}},\mathrm{ Nu}}$ based on UCS (Visualized from^53^ and revised from^22^).

The calculation of ${{{\varvec{\nu}}}_{{\varvec{N}}{\varvec{u}}} \, \text{,} {\varvec{E}}}_{{\varvec{N}}{\varvec{u}}} , {{\varvec{\sigma}}}_{{\varvec{c}},{\varvec{N}}{\varvec{u}}}$ is executed by performing the UCS test. This allows for the determination of Poisson’s ratio, Young’s modulus, and the peak compressive stress. Figure 5 illustrates the schematic calculation cycle for these parameters. Resultant axial stress–strain and axial stress-lateral strain curves provide the foundation for Eqs. (2) and (3), enabling the computation of E and the Poisson ratio^53^.

2 $$E=\frac{({\sigma }_{2}-{\sigma }_{1})}{{(\varepsilon }_{2}-0.000050)}$$

where E = modulus of elasticity, (GPa), ${\sigma }_{2}$ = Axial stress corresponding to 40% of UCS, (MPa), ${\sigma }_{1}$ = Axial stress corresponding to an axial strain, ${\varepsilon }_{1}$, of 50 millionths, (MPa), and ${\varepsilon }_{2}$= Axial strain produced by stress ${\sigma }_{2}$.

3 $$\nu =\frac{({\varepsilon }_{{l}_{2}}-{\varepsilon }_{{l}_{1}})}{({\varepsilon }_{2}-0.000050)}$$

where $\nu$ = Poisson’s ratio, ${\varepsilon }_{{l}_{2}}$ = Lateral strain at mid-height of the specimen produced by stress ${\sigma }_{2}$, and ${\varepsilon }_{{l}_{1}}$= Lateral strain at mid-height of the specimen produced by stress ${\sigma }_{1}$.

#### Validation process

The core of this validation process is centered around the fitness value calculation, which is fundamentally based on minimizing the Mean Squared Error (MSE) between the numerical macro-parameters and their corresponding experimental values. This approach ensures that the calibrated numerical parameters closely reflect the experimental data, thereby validating the effectiveness of our calibration methodology for CSCC.

##### Fitness value calculation of macro-parameters

Fitness value plays a pivotal role in guiding the GA's search through the solution space. In our calibration process, the fitness function aims at minimizing deviations between the numerical macro parameters produced by the PFC3D and the experimental benchmarks. A widely adopted approach for such optimization tasks is to compute the Mean Squared Error (MSE) between the numerical (predicted) and experimental (observed) values, which can be expressed as:

For each mix:

4 $$MSE=\frac{1}{n}{\sum_{i=1}^{n}({Numerical}_{i}-{Experimental}_{i})}^{2}$$

where *n* is the number of macro parameters.

5 $$=\frac{1}{3}\left({\left({\nu }_{Nu}-{\nu }_{Exp}\right)}^{2}+{\left({E}_{Nu}-{E}_{Exp}\right)}^{2}+{\left({\sigma }_{c, Nu}-{\sigma }_{c, Exp}\right)}^{2}\right)$$

The fitness value can then be inversely related to the MSE:

6 $$Fitness \,value=\frac{1}{1+MSE}$$

This formulation ensures that solution sets closely mirroring experimental records are assigned higher fitness values, guiding the GA toward optimal calibration. A higher fitness value, inversely related to the MSE, indicates a more accurate calibration. The fitness value for each numerical macro parameter is calculated through a GA in MATLAB, which also controls and runs the PFC3D simulations. This iterative validation process is applied until all 30 numerical macro parameters are accurately generated and validated against their corresponding experimental values, ensuring each parameter meets the predefined acceptance criteria.

##### Implicit calibration of micro-parameters through macro-level validation

In the intricate dynamics of numerical modeling, the interplay between micro-parameters (${\overline{{\varvec{K}}} }^{\mathbf{*}}{,{\varvec{K}}}^{\mathbf{*}}$**, **${\overline{{\varvec{E}}} }^{\mathbf{*}}$**, **${{\varvec{E}}}^{\mathbf{*}}$**, **$\overline{{{\sigma }}_{t}}$**,** $\overline{{\varvec{c}} })$ and their resultant macro parameters $({{{\varvec{\nu}}}_{{\varvec{N}}{\varvec{u}}} \, \text{,}\boldsymbol{ }\boldsymbol{ }\boldsymbol{ }{\varvec{E}}}_{{\varvec{N}}{\varvec{u}}}\boldsymbol{ },\boldsymbol{ }{{\varvec{\sigma}}}_{{\varvec{c}},{\varvec{N}}{\varvec{u}}})$ is foundational. By achieving a precise calibration of numerical macro parameters against experimental benchmarks, an implicit assertion is made: the underlying micro-parameters are likewise accurately calibrated. This is premised on the fact that the numerical macro parameters are direct manifestations of the micro-parameters set within the PFC3D environment. Thus, when macro outcomes closely align with experimental data, it validates the micro-parameter settings for each mix design.

## Results and discussions

### Validation of numerical macro-parameters

Following the meticulous calibration process facilitated by the integration of MATLAB's Genetic Algorithm with PFC3D, the numerically derived parameters— Poisson's ratio (*ν*), Young's modulus (*E*), and compressive strength (${{\varvec{\sigma}}}_{{\varvec{c}},}$)—were juxtaposed against their experimental analogs. Table 4 presents this comparative analysis in detail, underlining the proficiency of the model in reflecting empirical observations.

**Table 4:** Validated numerical macro-parameters (experimental results from^34^).

CSCC	Numerical results			Experimental results		
Mix ID	${\upnu }_{{\text{Nu}}}$	${{\text{E}}}_{{\text{Nu}}}$ (GPa)	${\upsigma }_{{\text{c}},{\text{Nu}}}$ (MPa)	${\upnu }_{{\text{Exp}}}$	${{\text{E}}}_{{\text{Exp}}}$ (GPa)	${\upsigma }_{{\text{Exp}}}$ (MPa)
Fa10	0.2035	34.73	53.81	0.2053	34.51	53.9
Blue5	0.2035	33.07	49.29	0.2057	33.1	49.6
Green5	0.2035	31.98	46.83	0.2046	32.12	46.7
Red5	0.2035	28.67	37.13	0.2039	28.78	37.5
Blue10	0.2035	31.98	46.41	0.2041	31.95	46.2
Green10	0.2036	31.31	43.96	0.2044	31.18	44.0
Red10	0.2035	26.47	31.54	0.2051	26.34	31.4
Blue15	0.2036	30.43	41.65	0.2049	30.31	41.6
Green15	0.2035	29.01	38.80	0.2055	29.2	38.6
Red15	0.2035	25.35	29.81	0.2037	25.74	30.0

**Figure 6:**
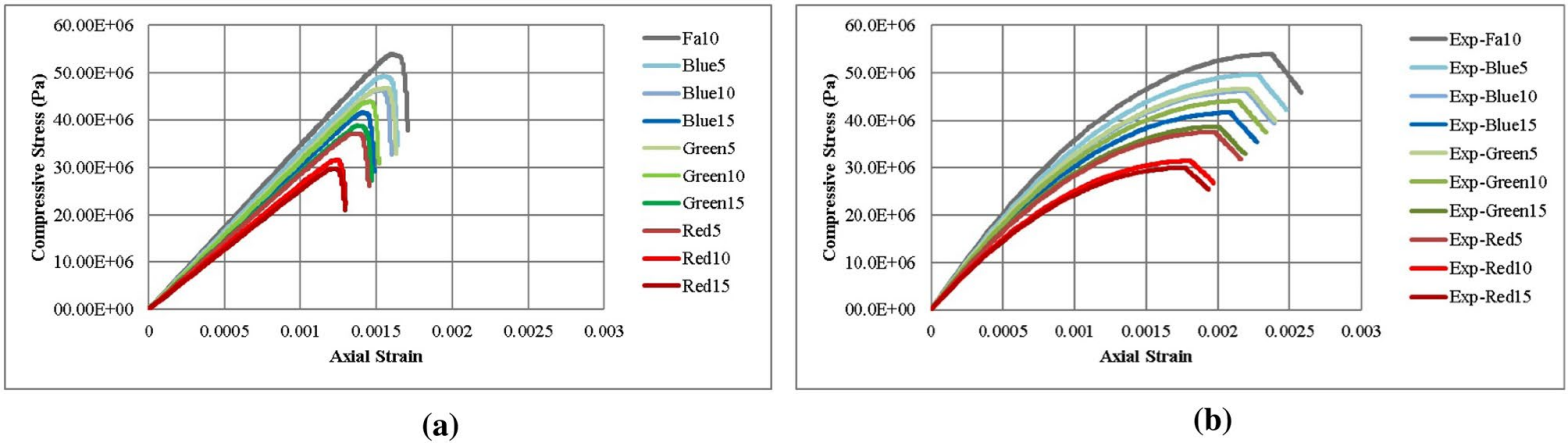
(**a**) Numerical stress–strain curves; (**b**) experimental stress–strain curves^34^; from UCS tests for all ten mix designs.

Figure 6a,b depict the numerical and experimental stress–strain curves for all ten mix designs, playing a crucial role in determining the E and ${{\varvec{\sigma}}}_{{\varvec{c}}}$ for each. Notably, the calibration process achieved high accuracy in capturing the peak stress values and Young's modulus, aligning with the primary objectives of this research. Upon detailed examination, while a degree of similarity is evident between the numerical and experimental stress–strain curves, certain differences are observed. These variations, particularly the reduced ductility observed in the numerical curves compared to the experimental data, underscore the precision in our modeling and point towards areas that merit further exploration, rooted in some different reasons.

In our DEM simulations, significant challenges arise in accurately capturing complexities such as aggregate crushing, grain boundary sliding, and the closing of pre-existing cracks within the bonded particle model^15^. These limitations particularly affect the stress–strain curves, leading to notable differences between the simulated and experimental results. Further contributing to these discrepancies is the simplification of particle (ball) size, shape, and form in our numerical model. Necessary for computational manageability, these simplifications do not entirely mirror the more intricate aggregate shapes and grading curves typical of actual CSCC. This issue is compounded by the variability in aggregate distribution and the extensive use of spherical balls (44,628) in the model, which complicates the precise matching of numerical results to experimental data. Insights from previous studies have highlighted that the free rotation of particles in the model can induce early yielding^54,55^.

Additionally, the use of the LPBM in the simulations tends to produce a more linear stress–strain curve, contributing to the observed variations in ductility and strain characteristics compared to the more complex responses seen in the experimental data. It is important to note that the study's primary focus was the development and validation of a calibration process using a multi-objective genetic algorithm, targeting micro–macro level mechanical properties with a particular emphasis on ${{\varvec{\sigma}}}_{{\varvec{c}}}$, E, and ν. While we endeavored to achieve high accuracy and equality for these three macro parameters, for other aspects such as ductility and strain at peak stress, a degree of similarity was deemed sufficient for the objectives of this research. Addressing the full spectrum of CSCC's mechanical behavior, especially the detailed non-linear responses and microcracking phenomena, was beyond the scope of this study. Future work will aim to refine the model to better align with experimental observations, taking into account the insights gathered and the limitations identified.

**Figure 7:**
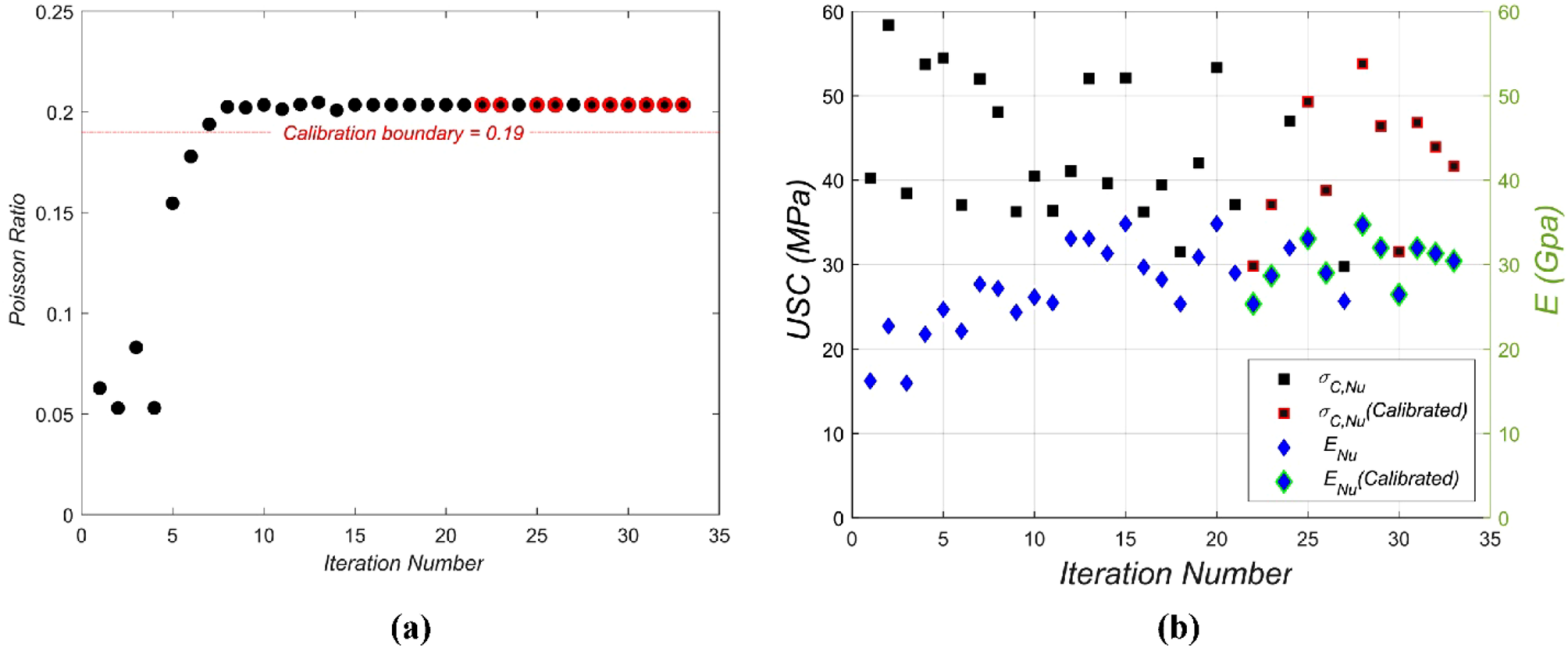
Iterative calibration progression of numerical macro-parameters. (**a**) Evolution of ${{\varvec{\upnu}}}_{\mathbf{N}\mathbf{u}}$ across iteration; (**b**) progression of ${\mathbf{E}}_{\mathbf{N}\mathbf{u}}$ and ${{\varvec{\upsigma}}}_{\mathbf{c},\mathbf{N}\mathbf{u}}$ across iterations.

Figure 7 showcases the dynamic nature of the iterative calibration process, unraveling the nuances of how various numerical macro-parameters evolve across 33 iterations.

In Fig. 7a, the trajectory of the ${{\varvec{\nu}}}_{{\varvec{N}}{\varvec{u}}}$ values over the calibration iterations is vividly illustrated. As the iterations unfurl, a discernible trend emerges where the Poisson ratio values consistently edge toward the experimental benchmarks. This alignment underscores the intrinsic linkage of the Poisson ratio to the micro-parameters ${\overline{{\varvec{K}}} }^{\mathbf{*}}{,{\varvec{K}}}^{\mathbf{*}}$. The calibration of the Poisson ratio is not an isolated endeavor but is inherently intertwined with the precise modulation of other numerical macro parameters. A calibration boundary, delineated at a Poisson ratio of 0.19, serves as a pivotal reference. Values ascending beyond this threshold resonate with the set criterion, echoing the intricate dance of calibration that juggles the macro and micro realms seamlessly. Turning to Fig. 7b, the iterative calibration journey of both the ${{\varvec{E}}}_{{\varvec{N}}{\varvec{u}}}$ and ${{\varvec{\sigma}}}_{{\varvec{c}},{\varvec{N}}{\varvec{u}}}$ is delineated. As the optimization forges ahead in a parallel manner, there is an incisive moment when the numerical values inch closer to one of the experimental touchstones. Upon sensing this convergence, the corresponding value is earmarked, and the calibration pivot shifts to the remaining mix design datasets. A pivotal realization dawns: the calibration of Young's modulus is intrinsically tethered to the modulation of the micro-parameters ${\overline{{\varvec{E}}} }^{\boldsymbol{*}},$ ${{\varvec{E}}}^{\boldsymbol{*}}$. Conversely, fine-tuning the compressive strength demands nuanced adjustments of the ${\overline{{\varvec{\sigma}}} }_{{\varvec{t}}}$**,** $\overline{{\varvec{c}} }$ micro-parameters, ensuring alignment with experimental insights.

**Figure 8:**
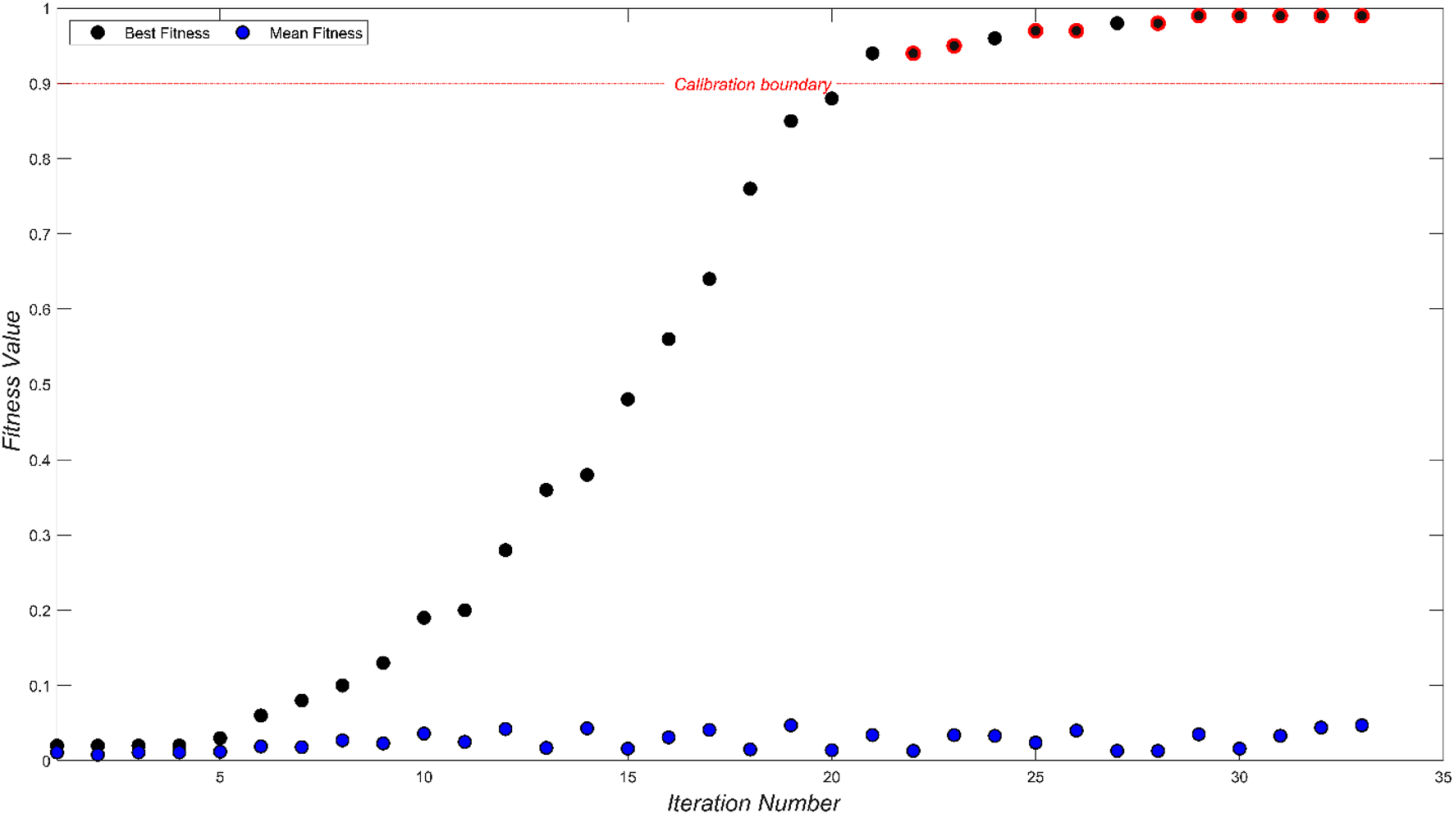
Fitness value evolution across 33 iterations for ten mix designs.

In Fig. 8, the evolution of fitness values across 33 iterations is vividly illustrated, casting light on the intricate calibration journey navigated by the Genetic Algorithm. This algorithm, fundamentally rooted in the quest for optimal micro-parameters, is driven to bridge the chasm between numerical simulations and experimental benchmarks. The dual trajectories portrayed in the figure—one spotlighting the best fitness values per iteration and the other tracing the mean fitness value—serve as a testament to this diligent expedition toward optimization.

The initial 20 iterations can be perceived as the algorithm's formative phase, where it meticulously hones its calibration instincts. Transitioning from this phase, the subsequent 13 iterations crystallize into a commendable achievement: the precise calibration of all ten mix designs. This progression, from foundational training to triumphant calibration, showcases the GA's adeptness in mastering the intricate calibration landscape.

Remarkably, throughout this journey, the fitness values for all ten mix designs consistently soar beyond the 0.94 threshold, a significant achievement when juxtaposed against the established benchmark of 0.9. Such unwavering attainment of elevated fitness scores, indicating a successful validation of the macro parameters, is emblematic of the algorithm's capability to adeptly traverse the solution space, refining its path towards optimal micro-parameter configurations that mirror experimental findings.

Collating these insights, it becomes unequivocally clear that the calibration methodology, bolstered by the Genetic Algorithm, stands as a meticulously curated approach in this study. This rigorous process evidently validates the macro parameters, as demonstrated by the pronounced alignment between the numerical and experimental stress–strain curves, while showing a degree of similarity, particularly in ductility and strain at peak stress, effectively validates the macro parameters. This alignment, further reinforced by the consistently high fitness values, underscores the model's capacity to capture the essential mechanical nuances, despite the slight variations. These results vouch for the calibration framework's robustness and precision, particularly in the accurate calibration of the three macro parameters—Uniaxial Compressive Strength, Modulus of Elasticity, and Poisson's Ratio. While the study does not encompass the full spectrum of CSCC's mechanical behavior, such as detailed non-linear responses and microcracking phenomena, the findings provide a solid foundation for future work aimed at refining the model to achieve even closer alignment with experimental observations.

### Calibrated micro-parameters

The micro-parameters tabled are a testament to the calibration's meticulousness, intertwined with the inherent behaviors of the CSCC mix designs (Table 5). A key observation across the dataset is the consistency in ${\overline{{\varvec{K}}} }^{\mathbf{*}}{,{\varvec{K}}}^{\mathbf{*}}$, both consistently hitting the upper boundary of 3.0. This outcome, while initially established as the maximum in the smart range strategy, underscores its importance. Literature reviews and our current findings converge on this value, highlighting its pivotal role in representing the material's intrinsic characteristics.

**Table 5:** Calibrated micro-parameters for ten CSCC mix designs.

CSCC	Parallel bond micro-parameters					
Mix ID	${\overline{{\text{K}}} }^{*}$	${{\text{K}}}^{*}$	${\overline{{\text{E}}} }^{*}$ (GPa)	${{\text{E}}}^{*}$ (GPa)	${\overline{\upsigma } }_{{\text{t}}}$ (MPa)	$\overline{c }$ (MPa)
Fa10	3.0	3.0	15.75	15.75	8.1	10.0
Blue5	3.0	3.0	15.00	15.00	7.4	9.0
Green5	3.0	3.0	14.50	14.50	7.0	8.8
Red5	3.0	3.0	13.00	13.00	5.5	7.0
Blue10	3.0	3.0	14.50	14.50	7.0	8.4
Green10	3.0	3.0	14.20	14.20	7.0	7.0
Red10	3.0	3.0	12.00	12.00	5.0	5.0
Blue15	3.0	3.0	13.80	13.80	6.4	7.0
Green15	3.0	3.0	13.15	13.15	5.8	7.0
Red15	3.0	3.0	11.50	11.50	4.6	5.0

The Young’s moduli, ${\overline{{\varvec{E}}} }^{\boldsymbol{*}},$ ${{\varvec{E}}}^{\boldsymbol{*}}$, present values within the defined calibration bounds. Their range, although broad, aligns with the experimental trend. These values represent the stiffness attributes of the CSCC, and their calibration provides insights into the material's resistance to elastic deformation. The trend in the compressive and tensile strengths, embodied by ${\overline{\upsigma } }_{{\text{t}}}$ and $\overline{c }$ respectively, offers a clear indication of the CSCC's ability to withstand stresses. They stay within the anticipated bounds, and their calibrated values are a testament to the algorithm's capability to match real-world experimental data closely.

It is essential to note that the calibration process, underpinned by the Genetic Algorithm, was not an arbitrary pursuit. The initial bounds, set based on a literature-backed smart range strategy, played a crucial role. The algorithm's journey, from initial bounds to the calibrated values, reflected its inherent ability to navigate the solution space effectively. The results, especially the attainment of the upper bound for ${\overline{{\varvec{K}}} }^{\mathbf{*}}{,{\varvec{K}}}^{\mathbf{*}}$, signify the algorithm's finesse in aligning with real-world expectations and the validity of the set bounds.

Pigments' role in the entire calibration process warrants special attention. Their introduction, especially in substantial proportions, has a nuanced effect on the CSCC's micro-parameters. While it would be clear that the pigments influence the mechanical properties of the CSCCs, the calibrated micro-parameters encapsulate these effects. The values, particularly of ${\overline{{\varvec{\sigma}}} }_{{\varvec{t}}} , \overline{{\varvec{c}} }$, ${\overline{{\varvec{E}}} }^{\boldsymbol{*}}$ and ${{\varvec{E}}}^{\boldsymbol{*}}$ are inherently reflective of the composite structure of the CSCC, including the pigments. This holistic representation ensures that the cumulative impact of the mix design, inclusive of the pigments, finds its voice in the calibrated micro-parameters.

In summation, the presented calibrated micro-parameters, when compared to the set bounds and experimental data, emphasize the robustness of the calibration methodology. The tabled values offer a holistic understanding, ensuring the influence of components like pigments is effectively integrated, setting a solid foundation for future endeavors in concrete simulations.

In this study, our calibration process primarily centered on the UCS test to align the micro-parameters in the DEM model with the macro-level mechanical properties of hardened CSCC. This focus was guided by the objective to simulate and understand the long-term structural behavior of CSCC. We recognize the significance of early-age properties such as viscosity, yield stress, and flow characteristics (typically assessed through slump flow, L-box, V-funnel, and rheometer tests) in distinguishing SCC from conventional concrete. These properties are crucial in evaluating the workability and flowability of SCC in its fresh state, ensuring ease of placement and adequate filling capacity without segregation. While our current study did not directly simulate these early-age properties, the calibrated model based on UCS provides a robust foundation for future expansions. Such expansions could potentially include the simulation of SCC's distinctive rheological properties, offering a comprehensive understanding of both its hardened and fresh-state behaviors. The inclusion of these aspects in future work would enhance the applicability of the model, particularly in scenarios where the fresh-state properties of SCC/CSCC are as critical as its hardened state.

The multi-objective genetic algorithm's versatility in calibration applications is profoundly illustrated by its successful implementation in diverse fields, extending well beyond the confines of our current study. Notably, its potential in soil and rock mechanics is significant, especially in contexts where triaxial and biaxial tests are prevalent for calibrating numerical models. One study in the realm of cohesive bulk materials, particularly coal, employed the Non-dominated Sorting Genetic Algorithm (NSGA) to optimize parameters of an Elasto-Plastic Adhesive contact model. This approach effectively captured varied stress states and history dependencies in coal, aligning simulations closely with experimental shear stress measurements. Another research project unveiled a universal framework for calibrating microscopic properties in granular materials, utilizing the NSGA-II. This framework, designed for industrial-scale applications and adaptable to all DEM simulation setups, successfully balanced model accuracy with computational efficiency, highlighting the use of Pareto dominance principles to manage trade-offs between conflicting objectives. These instances not only underscore the genetic algorithm's adaptability across different materials and simulation challenges but also emphasize its potential to contribute to a wide range of industrial applications and computational modeling fields, paving the way for future research and advancements.

## Conclusion

In the advanced realm of structural engineering research, the intricate behaviors of CSCC have been consistently explored. This investigation was meticulously designed to shed light on these nuances, aligning sophisticated numerical simulations with experimental findings more closely than ever before.

The core of this study revolved around the precision calibration of micro-parameters across ten distinctive CSCC mix designs. This was achieved by leveraging MATLAB's Genetic Algorithm in conjunction with PFC3D software, forging an innovative calibration pathway. This union birthed an automated, multi-objective optimization process, transcending traditional calibration methods and aligning simulations with the subtleties observed in real-world CSCC experiments.

Key contributions and insights from this study include:

1. The pioneering methodology, integrating MATLAB's Genetic Algorithm with PFC3D, introduced an automated and systematic approach to multi-objective optimization for CSCC. This process significantly improves upon traditional trial-and-error methods, enabling precise calibration of micro-parameters for ten distinct CSCC mix designs concurrently. This orchestrated calibration not only augmented precision but also streamlined computational efficiency. The genetic algorithm has proven to be both versatile and robust, demonstrating its effectiveness in a wide range of calibration applications across various material behaviors and industrial contexts.
2. The successful validation of the three numerical macro parameters—Uniaxial Compressive Strength, Modulus of Elasticity, and Poisson's Ratio—in our study directly led to the calibration of the associated micro-parameters. This outcome, supported by consistently high fitness values exceeding the 0.94 threshold, not only validates the macro parameters but also ensures the accurate calibration of micro-parameters, demonstrating the precision and reliability of our methodology.
3. The inclusion of pigments in the CSCC mix, while elucidating certain behaviors, also accentuated the complexity of the calibration challenge. However, their influences were adeptly encapsulated within the calibrated micro-parameters, offering a holistic insight into the mechanical attributes of the mixes.

Nevertheless, inherent constraints and challenges were observed. Utilizing PFC3D for concrete simulations, while innovative, may have limitations in capturing certain granular behaviors specific to concrete. Similarly, while the Genetic Algorithm offers a robust calibration mechanism, its efficacy is intrinsically tied to the defined parameter bounds, suggesting potential areas for further refinements and broader applications.

In essence, this research has marked a substantial leap in the domain of concrete simulations. It underscores the merits of juxtaposing advanced computational tools with rigorous experimental data to derive profound insights. Future research trajectories should consider deeper exploration into the long-term behaviors of CSCC mixes under diverse conditions, continually refining our understanding and applications in the realm of structural engineering.

## Data Availability

The data presented in this study is available upon corroborated re-quest from the corresponding author.

## Abbreviations

SCC: Self-compacting concrete
CSCC: Colored Self-compacting concrete
${\text{DEM}}$: Discrete element method
${\text{PFC}}$: Particle flow code
GA: Genetic algorithm
MOGA: Multi-objective genetic algorithm
UCS: Uniaxial compressive strength
PBCM: Parallel bonded contact model
PBPM: Parallel bonded particle model
LPBM: Linear parallel bond model
*H*: Model height
*W*: Model weight
V: The velocity of loading platen
${\sigma }_{c,Exp}$: Experimental peak compressive stress
${E}_{Exp}$: Experimental Young module
${\nu }_{Exp}$: Experimental Poisson ratio
${\sigma }_{c,Nu}$: Numerical peak compressive stress
${E}_{Nu}$: Numerical Young module
${\nu }_{Nu}$: Numerical Poisson ratio
$${R}_{min}$$: Minimum radius
$\frac{{R}_{max}}{{R}_{min}}$: Maximum to Minimum radius ration
$$\rho$$: Ball densiy
${g}_{i}$: Installation bond gap
$$\phi$$: Friction angle
$$\mu$$: Friction coefficient
${K}^{*}$: Normal-to-shear stiffness ratio
${\overline{K} }^{*}$: Parallel bond normal-to-shear stiffness ratio
$${E}^{*}$$: Effective modulus
${\overline{E} }^{*}$: Parallel bond effective modulus
$\overline{{{\sigma }}_{t}}$: Parallel bond tensile strength
$\overline{c }$: Parallel bond cohesion
Pb: Parallel bond
